# Non-invasive assessment of the physiological role of leaf aerenchyma in *Hippeastrum* Herb. and its relation to plant water status

**DOI:** 10.1007/s00425-022-03930-2

**Published:** 2022-06-24

**Authors:** Paulo Cabrita

**Affiliations:** IAPN—Institute of Applied Plant Nutrition, Georg-August-University Göttingen, Carl-Sprengel-Weg 1, 37075 Göttingen, Germany

**Keywords:** Aerenchyma pressure, Amaryllidaceae, Gas exchange measurements, Leaf patch clamp pressure probe, Plant–water relations, Turgor pressure

## Abstract

**Main conclusion:**

The leaf patch clamp pressure probe combined with gas exchange measurements provides a non-invasive approach for measuring leaf aerenchyma pressure and study its physiological role in plants.

**Abstract:**

The non-invasive leaf patch clamp pressure probe (LPCP) measures the output pressure, *P*_p_, in response to the pressure applied by two magnets clamped to a leaf. In many plant species, it has been observed that the diel pattern of *P*_p_ follows the changes in the leaf turgor pressure reversely. The genus *Hippeastrum* comprises 143 species and many hybrids and cultivars of high economic value within Amaryllidaceae. Their leaves are characterized by the presence of aerenchyma composed of lacunae, running throughout the leaf and composing most of the mesophyll volume. In *Hippeastrum*, the diel changes of the LPCP output pressure are the reverse of that observed on the air pressure in the leaf aerenchyma, *P*_a_, which depends on the changes in the leaf vapor pressure occurring during photosynthesis. A theoretical model is proposed and confirmed experimentally by LPCP and gas exchange measurements. The output pressure, *P*_p_, in *Hippeastrum* can be related to the plant water status through the gas exchange processes that occur during photosynthesis. Considering the natural habitats of *Hippeastrum* species, these results agree with the physiological role of leaf aerenchyma in facilitating gas transport and light scattering in leaves, thus contributing to the photosynthetic efficiency of these plants under adverse environments. A second, but supplemental, interpretation of the LPCP output pressure, *P*_p_, when applied on species in which the aerenchyma constitutes most of the mesophyll volume is presented.

## Introduction

The leaf patch clamp pressure probe (LPCP) is a non-invasive plant-based system that measures the output pressure, *P*_p_, in response to the pressure applied by two magnets clamped to a leaf, *P*_clamp_ (Zimmermann et al. 2008, 2013). The output pressure, *P*_p_, is a power function of the turgor pressure, *P*_c_, of the clamped leaf patch given by (Zimmermann et al. 2008):

1 $${P}_{\mathrm{p}}={\left(\frac{b}{{aP}_{\mathrm{c}}+b}\right)}^\frac{1}{a}{{F}_{a}P}_{\mathrm{clamp}}.$$

Due to the hydraulic continuity with the surrounding leaf tissue, the leaf patch can be used as a sensing element of the plant water status and to study indirectly physiological processes that depend on the turgor pressure. The output pressure, *P*_p_, depends on the hydraulic conductivity of the leaf patch in transmitting the pressure applied by the magnets, *P*_clamp_, throughout all its cells (Zimmermann et al. 2008, 2010, 2013; Ehrenberger et al. 2012b). The bigger the leaf patch volume, the longer the transmission pathway and the higher its resistance; hence, the smaller its conductivity, to transmitting pressure. Therefore, the output pressure, *P*_p_, will be smaller due to the higher attenuation of the applied pressure. The leaf patch volume and its resistance to transmit the pressure applied by the magnets, *P*_clamp_, depend on the different leaf components (e.g., cell walls, protoplasts, cuticle, intercellular air spaces). In leaves where the mesophyll is mostly filled with turgid protoplasts, changes in the cell volume, due to water movement, are determined by the elastic properties of the cell walls that limit them, namely, through their volumetric elastic modulus, *ε*, (Broyer 1952; Philip 1958):

2 $$\upvarepsilon =V\frac{\partial {P}_{\mathrm{c}}}{\partial V}.$$

Plant cells experience bigger changes in their volumes for smaller values of *ε* (i.e., more elastic cell walls) for a given increase in turgor pressure ∂*P*_c_. The volumetric elastic modulus is a measure of how resistant plant cells are to compression. Consequently, as they slightly expand by increasing their turgor pressure, due to osmotic water uptake, the overall volume of the leaf patch will increase along with its resistance to transmit *P*_clamp_. Thus, the attenuation of *P*_clamp_ will also increase, leading to smaller values of the output pressure, *P*_p_. Considering the dependence of *ε* on temperature (*T*) and turgor pressure (Murphy and Ortega 1995), Zimmermann et al. (2008) concluded that the attenuation of *P*_clamp_ is a function of the volume, compressibility of the cell walls, and hydraulic conductivity of the leaf patch, expressed by parameters *a* and *b*, as well as the compressibility of the turgor-independent components of the leaf (e.g., cuticle) and the silicone membrane of the pressure sensor itself, both combined in the attenuation factor F_a_ Eq. (1). Therefore, the diel pattern of *P*_p_ will be reversed to that of *P*_c_ Eq. (1) (Zimmermann et al. 2008).

Equation (1) has been observed on many plant species, from big plants and trees, e.g., banana (*Musa acuminata* Colla) (Zimmermann et al. 2010), *Banksia menziesii* R.Br. (Bader et al. 2014), chestnut vine (*Tetrastigma voinierianum* (Baltet) Gagnep.) (Zimmermann et al. 2008), *Eucalyptus* spp. (Rüger et al. 2010), grape vine (*Vitis vinifera* L.) (Westhoff et al. 2009); maize (*Zea mays* L.) (Riboldi et al. 2016), oak (*Quercus rubor* L.) (Ehrenberger et al. 2012a), olive tree (*Olea europaea* L.) (Fernández et al. 2011), to smaller plants, e.g., *Arabidopsis thaliana* (L.) Heynh. (Ache et al. 2010), rapeseed (*Brassica napus* L.) (Kant et al. 2014), tomato (*Solanum lycopersicum* L.) (Lee et al. 2012), wheat (*Triticum aestivum* L.) (Bramley et al. 2013), under normal and low turgor pressure as well as plasmolysis (Ehrenberger et al. 2012b). The intercellular air spaces in all species studied so far are ubiquitously and uniformly distributed throughout the mesophyll. Their total volume is much smaller than that of chlorenchyma and associated cells so that its overall attenuating effect on *P*_clamp_ is small and reflected through the attenuation factor *F*_a_ Eq. (1). The attenuation factor *F*_a_ has been observed as constant for turgid tissues (Zimmermann et al. 2008, 2010) but significant and variable under low turgor pressure and plasmolytic states (Ehrenberger et al. 2012b). Therefore, one may ask how does the output pressure, *P*_p_, vary and what physiological parameters can be related to it in species in which the aerenchyma constitutes most of the leaf volume, namely, being specifically arranged in big distinct structures, e.g., canals or lacunae (Raven 1996; Evans 2003). In these species, a big fraction of the leaf patch volume will be mostly composed of large intercellular spaces filled with saturated air under normal physiological conditions (Sharkey 1985; Salisbury and Ross 1991; Taiz et al. 2014; Drake et al. 2019).

Leaves containing big areas of aerenchyma are found in the genus *Hippeastrum* of the family Amaryllidaceae (Arroyo and Cutler 1984; Meerow and Snijman 1998). This genus comprises 143 entirely New World species (WFO 2021) and over 600 intrageneric hybrids and cultivars of perennial herbaceous hysteranthous plants, with large fleshy tunicate bulbs, long broad leaves, and big showy flowers. Its centers of diversity lie in eastern Brazil and the eastern slopes and adjacent foothills of central southern Andes of Peru, Bolivia, and Argentina, while some species extend further north to Mexico and the West Indies (Meerow 2004, 2009). This genus has been intensely bred and cultivated for its colorful flowers, reflecting its high ornamental value within Amaryllidaceae. Its species are found in a wide range of habitats, with many living underbrush while others preferring full sun conditions, from flood areas to dry locations. The leaves of *Hippeastrum* are characterized by the presence of aerenchyma composed of big interfascicular air cavities, of lysigenous origin, called lacunae, running throughout the whole length of the leaf parallel to the midrib, reaching both the endodermis and the chlorenchyma of the abaxial and adaxial leaf surfaces, composing up to 80% of the mesophyll volume (Arroyo and Cutler 1984; Meerow and Snijman 1998; Alves-Araújo and Alves 2005, 2007; Alves-Araújo et al. 2012; Marques 2015; Zhou et al. 2012).

A second, but supplemental, interpretation of the leaf patch clamp output pressure, *P*_p_, is here presented. The effect of specific leaf aerenchyma structures on the output pressure, *P*_p_, will be shown, using specimens of *Hippeastrum* ‘Red Lion’ as model plants. Results will be then compared with those of banana (*Musa acuminata* Colla), a species studied in previous works. To that end, a theoretical model is described and validated experimentally, in which knowledge of the leaf structure is shown to be crucial to best interpret the main factors behind the leaf patch attenuation of *P*_clamp_. In species in which the aerenchyma constitutes most of the leaf volume, e.g., *Hippeastrum*, the output pressure, *P*_p_, can be still related to the leaf turgor pressure and plant water status by the gas exchange processes that occur within the aerenchyma, which depend on the photosynthetic activity.

## Materials and methods

### Plants

Two 4-year-old specimens of *Hippeastrum* ‘Red Lion’ cultivar and one 2-year-old banana plant (*Musa acuminata* Colla (AAA group) ‘Dwarf Cavendish’) were grown in 6 l pots, using the soil mix, designed for ornamental plants, Fruhstorfer Erde Typ T Struktur 1b (HAWITA GRUPPE GmbH, Vechta, Germany), watered and fertilized regularly with a N–P–K ratio of 2.5–1–2 (Universal-Flüssigdünger, Schmees GmbH, Twistringen, Germany) in the greenhouse at IAPN—Institute of Applied Plant Nutrition, Göttingen, Germany. Weather parameters were continuously monitored (Web-Thermo-Hygrobarograph, Wiesemann and Theis GmbH, Wuppertal, Germany), under a 16/8-h light/dark regime, supplied by high pressure sodium-vapor lamps (Philips MASTER AGRO 400 W). The photon flux density varied between 150 and 1400 µmol m^−2^ s^−1^ during the light period at 1 m above ground. Water deficit was imposed by withholding watering. Transient and rapid changes of the ambient parameters were performed by opening and closing the windows for specific periods.

### Microscopy

For the microscopy analysis, 1 cm long samples of fully expanded leaves of *Hippeastrum* plants, cut halfway along the leaf length, were fixed in formalin–acetic acid–alcohol (FAA 50) (Johansen 1940) under vacuum for 48 h, to best extract the air trapped within the tissues, and preserved afterwards in 20% ethanol. Fixed samples were transversely sectioned, 80–100 μm thick, using a KD-2950 Cryostat Microtome (Kedi Instrumental Equipment Co. Ltd., Jinhua, China). The sections were then clarified using 50% sodium hypochlorite (Marques 2015) and stained with Toluidine Blue O (Peterson et al. 2008). Photographs were taken using a Canon EOS 600D camera mounted on a Primo Star light microscope (Carl Zeiss Microscopy, Oberkochen, Germany). The aerenchyma lacunae were analyzed using the ImageJ software, v. 1.53q, and its thickness expressed relative to the mesophyll thickness.

### Leaf gas exchange measurements

Photosynthetic parameters [CO_2_ net assimilation rate (*A*), stomatal water vapor conductance (g_H2O_), leaf temperature (*T*_Leaf_), transpiration rate (*E*), and leaf-to-air vapor pressure deficit (VPD)] were measured with a portable open gas exchange fluorescence system GFS-3000-C equipped with a high-precision CO_2_/H_2_O infrared gas analyzer and a standard measuring head 3010-S (Heinz Walz GmbH, Effeltrich, Germany). Plant leaves were enclosed in a 4 cm^2^ cuvette with its parameters (temperature, relative humidity, light) set to follow the ambient conditions. Flow rate was set to 750 µmol s^−1^ and measurements commenced with a 1-min sampling interval, following stability in the cuvette, achieved in 2–3 min after enclosing the leaf in the cuvette approximately. The leaf aerenchyma water vapor pressure, VP_Leaf_, was obtained from the definition of leaf-to-air vapor pressure deficit (VPD):

3 $${\mathrm{VP}}_{\mathrm{Leaf}}=\mathrm{VPD}+{\mathrm{VP}}_{\mathrm{air}},$$

where VP_air_ is the outside air vapor pressure given by:

4 $${\mathrm{VP}}_{\mathrm{air}}=\frac{{\mathrm{RHVP}}_{\mathrm{sat}}}{100},$$

with RH being the relative humidity of the ambient air and VP_sat_ the ambient air saturation pressure (Alduchov and Eskridge 1996). Whenever observed, stomatal oscillations were counted, and their average period determined per light phase.

### Leaf patch clamp pressure probe (LPCP)

The output pressure, *P*_p_, was measured using a set of 15 leaf patch clamp pressure probes and associated components for telemetric and mobile network-based data transfer to the internet with a 1-min sampling interval (YARA ZIM Plant Technology GmbH, Hennigsdorf, Germany). To assure uniform contact with the leaf and measure the output pressure, *P*_p_, magnets were placed avoiding thick veins, with the magnet containing the pressure sensor applied on the abaxial leaf surface unless specified otherwise. As with stomatal oscillations, fluctuations in the output pressure, *P*_p_, were counted and their average period determined per light phase.

### The output pressure, *P*_p_, of leaves in which aerenchyma constitutes most of leaf volume: theoretical model

The leaf patch clamp pressure output pressure, *P*_p_, equals the input pressure, *P*_in_, experienced by the cells in a leaf patch, only if the pressure applied by the magnets, *P*_clamp_, is transmitted lossless to all affected cells. Due to the compressibility and deformability of the silicone membrane of the pressure sensor as well as that of the cuticle, there are always losses of *P*_clamp_ (Zimmermann et al. 2008, 2010). That is, *P*_in_ is always smaller than *P*_clamp_ so that only a small fraction of *P*_clamp_ affects the leaf patch cells. *P*_clamp_ is thus attenuated by the resistance of the leaf tissue to being compressed. Hence, an attenuation factor, *F*_a_, always smaller than 1 and dependent on the individual mechanical properties of the leaf and the compressibility of the silicone membrane of the pressure sensor, is defined as (Zimmermann et al. 2008):

5 $${F}_{\mathrm{a}}=\frac{{P}_{\mathrm{in}}}{{P}_{\mathrm{clamp}}}.$$

Under normal physiological conditions, F_a_ can be considered constant (and the changes in the leaf thickness negligible) in turgid leaf tissues compressed by *P*_clamp_ (Ehrenberger et al. 2012a, b). Therefore, for a constant *P*_clamp_, *P*_in_ will also be constant Eq. (5), and the output pressure, *P*_p_, will be then determined by the leaf patch hydraulic properties in transmitting *P*_in_ throughout. These properties are reflected in what is called the leaf patch transfer function, *T*_f_, which depends on the leaf patch volume, *V*, being compressed. The transfer function *T*_f_ reflects the transmission of pressure by determining the fraction of *P*_in_ measured by the LPCP probe. *T*_f_ is therefore dimensionless and varies between 0 and 1 (Zimmermann et al. 2008). Using Eq. (5), *P*_p_, will, thus, be given by:

6 $${P}_{\mathrm{p}}={T}_{\mathrm{f}}\left(V\right){P}_{\mathrm{in}}={T}_{\mathrm{f}}\left(V\right){{F}_{\mathrm{a}}P}_{\mathrm{clamp}}.$$

In species in which the leaf volume is mostly occupied by aerenchyma composed of lacunae (e.g., *Hippeastrum*) practically filled with saturated air^1^ (Salisbury and Ross 1991; Taiz et al. 2014), running parallel to the midrib and reaching both the abaxial and adaxial surfaces (Arroyo and Cutler 1984; Alves-Araújo and Alves 2005; Alves-Araújo et al. 2012; Zhou et al. 2012; Marques 2015), the likelihood of having a leaf patch mostly made of aerenchyma lined by smaller chlorenchyma cells is quite high. In these species, the leaf patch volume will be most likely composed of saturated air-filled space. Therefore, one can consider that *T*_f_ depends on the volume of the lacunae being compressed, *V*_a_. Accordingly, the bigger the volume of the leaf patch, *V*_a_, the bigger the pathway and its resistance to transmit, *P*_in_, throughout and, hence, the smaller the values of *T*_f_ will be. In these species, let us consider *T*_f_ of the form:

7 $${T}_{\mathrm{f}}=\frac{k}{{V}_{\mathrm{a}}},$$

where *k* is an undetermined constant. From Eq. (7), one has that:

8 $${T}_{\mathrm{f}}=-{\left(\frac{\partial {T}_{\mathrm{f}}}{\partial {V}_{\mathrm{a}}}\right)}_{T}{V}_{\mathrm{a}},$$

where the suffix *T* means that, under normal physiological conditions, one neglects the changes in *T*_f_ due to temperature. This means that, in these cases, the changes in the compressibility of the leaf patch as well as that of the silicone membrane of the pressure sensor are too small to affect the transmission pressure. Under normal ambient conditions, the saturated air in the leaf aerenchyma (Sharkey 1985; Salisbury and Ross 1991; Drake et al. 2019; Taiz et al. 2014) can be assumed as behaving like an ideal gas (Çengel 2007). Therefore, at a given temperature *T*, any small changes in the volume of leaf aerenchyma will depend on the pressure of the saturated air in the aerenchyma, *P*_a_. That is:

9 $${\left(\frac{\partial {P}_{\mathrm{a}}}{\partial {V}_{\mathrm{a}}}\right)}_{T}=\frac{{K}_{\mathrm{T}}}{{V}_{\mathrm{a}}},$$

where *K*_T_ is the isothermal bulk modulus of air (Çengel 2007). The isothermal bulk modulus of an ideal gas is equal to its pressure, therefore, *K*_T_ = *P*_a_. Thus, combining Eqs. (8) and (9) and considering the ideal gas law, one obtains:

10 $$\frac{\mathrm{d}{T}_{\mathrm{f}}}{{T}_{\mathrm{f}}}=-\frac{\mathrm{d}{P}_{\mathrm{a}}}{{P}_{\mathrm{a}}}.$$

From which, being a separable differential equation, one obtains after integration:

11 $${T}_{\mathrm{f}}=\frac{\alpha }{{P}_{\mathrm{a}}}$$

and the output pressure *P*_p_ Eq. (6) will, thus, be given by:

12 $${P}_{\mathrm{p}}=\frac{\mathrm{\alpha }{F}_{\mathrm{a}}}{{P}_{\mathrm{a}}}{P}_{\mathrm{clamp}},$$

where *α* is an undetermined constant. Hence, in *Hippeastrum* leaves, the changes of the output pressure, *P*_p_, Eq. (6) with time are caused by changes in the leaf aerenchyma pressure, *P*_a_. *P*_p_ is thus inversely proportional to the aerenchyma air pressure, *P*_a_ Eq. (12).

When stomata open, the internal leaf atmosphere and the air outside form a continuum. Due to the extremely high surface-area-to-volume ratio of the leaf intercellular spaces, as the internal leaf surface can be 7–50 times the external leaf area (Carriqui et al. 2020), water vapor equilibrium inside the leaf is reached very rapidly. As the internal leaf atmosphere is at water potential equilibrium with the cell wall surfaces from which water evaporates, the air inside the leaf is always saturated basically. Therefore, through the open stomata a water vapor concentration gradient is kept between the leaves and the unsaturated air outside favoring the evaporation of water from the thin aqueous film lining the mesophyll cell walls facing the leaf intercellular air spaces. After water has evaporated from the cell wall surface into the intercellular air space, diffusion is the primary means of any further movement of water out of the leaf through the stomata. Transpiration is, thus, controlled by the concentration gradient of water vapor between the leaf intercellular air spaces and the air outside and the diffusional resistance of this pathway. With the daily increase in temperature, the moisture holding capacity of air increases, decreasing the relative humidity of the air outside and increasing the water vapor concentration in the leaf intercellular air spaces concomitantly. Therefore, the water vapor gradient between the leaves and the outside air increases. Due to the increase in the leaf water vapor concentration with temperature, there is a subsequent build up in the leaf intercellular air spaces vapor pressure that increases the local air pressure. This buildup in pressure of the saturated air inside leaves makes the aerenchyma air pressure, *P*_a_, greater than the ambient pressure surrounding them, as the ambient air is not saturated. Thus, a positive leaf-to-air vapor pressure deficit, VPD, Eq. (3) between the leaves and the surrounding outside air is kept, allowing water vapor to be released to the air outside by diffusion through stomata. Under these conditions, the output pressure, *P*_p_, decreases throughout the day as the aerenchyma air pressure, *P*_a_, increases due to transpiration Eq. (12). In the afternoon, the ambient temperature decreases, thus decreasing the moisture holding capacity of air. Consequently, *P*_a_ decreases, as the amount of water released from the mesophyll and needed to keep the air saturated in the leaf intercellular air spaces will be less than that needed when the temperature was higher earlier in the day. Therefore, transpiration decreases due to the decrease in the leaf-to-air vapor pressure deficit, VPD, Eq. (3) as the ambient relative humidity increases. The aerenchyma air pressure, *P*_a_, should, thus, reach its lowest once stomata close and transpiration ceases at night, when temperature reaches its lowest. Hence, as stomata close, *P*_p_ increases and will be kept higher during the night than it was during the day. Consequently, *P*_p_ presents the reversed pattern of the diel changes observed on the aerenchyma air pressure, *P*_a_, in *Hippeastrum* leaves. That is, from Eq. (12) one has that:

13 $$\frac{\partial {P}_{\mathrm{p}}}{\partial t}=-\frac{{P}_{\mathrm{p}}}{{P}_{\mathrm{a}}}\frac{\partial {P}_{\mathrm{a}}}{\partial t}.$$

Considering that the air in the leaf intercellular air spaces, including the lacunae of the leaf aerenchyma, is a mixture of several components (Çengel, 2007) as the ambient air surrounding the leaf, aerenchyma pressure, *P*_a_, is given by:

14 $${P}_{\mathrm{a}}={\mathrm{VP}}_{\mathrm{Leaf}}+\upxi ,$$

where VP_Leaf_ is the leaf aerenchyma vapor pressure Eq. (3) and *ξ* is the sum of the respective aerenchyma partial pressures of all components of air except water, e.g., CO_2_, O_2_, N_2_. Thus, substituting *P*_a_ Eq. (14) into Eq. (12), one obtains:

15 $${\mathrm{VP}}_{\mathrm{Leaf}}=\frac{\mathrm{\alpha }{F}_{\mathrm{a}}{P}_{\mathrm{clamp}}}{{P}_{\mathrm{p}}}-\upxi .$$

The main factor contributing to changes of the leaf aerenchyma pressure, *P*_a_, is the change in leaf water vapor concentration due to transpiration, i.e., VP_Leaf_ Eq. (3).

## Results

### The diel patterns of the output pressure, ***P***_p_, in banana and ***Hippeastrum*** are opposite

**Fig. 1:**
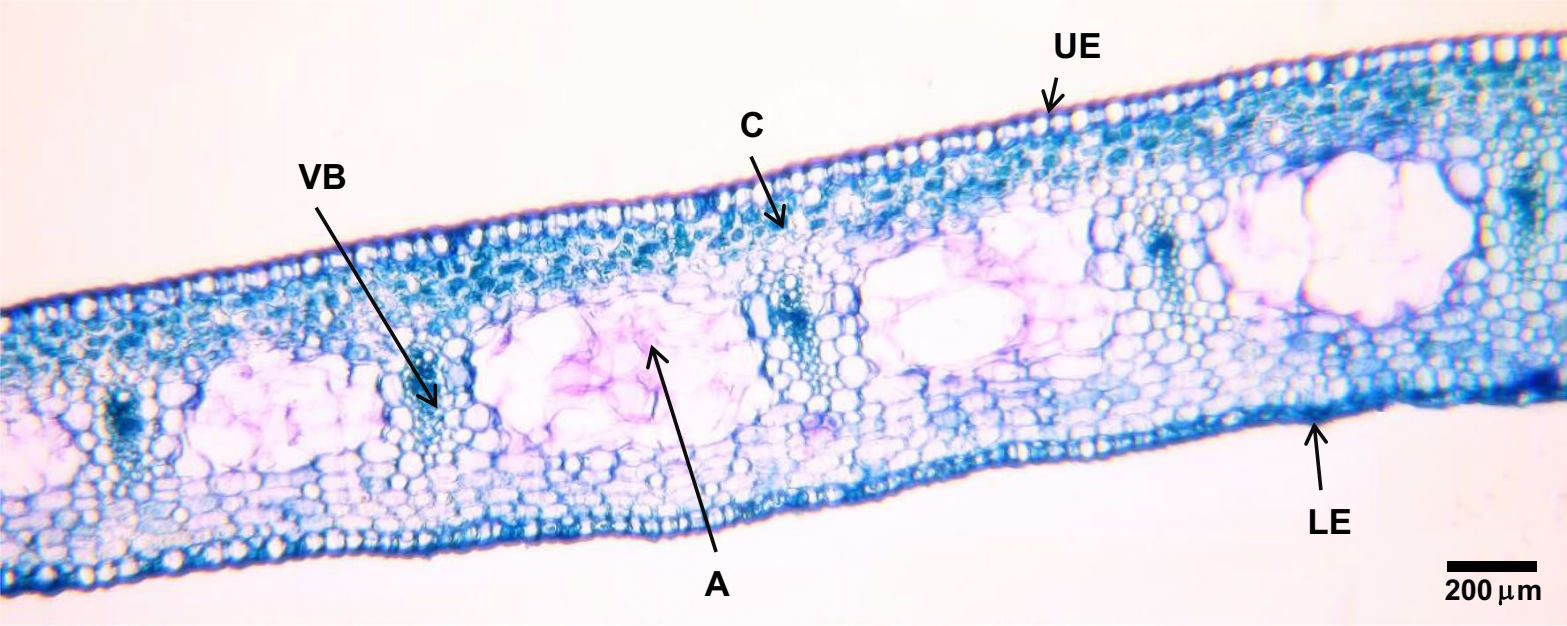
*Hippeastrum* leaf cross section micrograph (40✕). A, aerenchyma lacuna, interspersing the vascular bundles; C, chlorenchyma; LE, lower (abaxial) epidermis; UE, upper (adaxial) epidermis; VB, vascular bundle

**Fig. 2:**
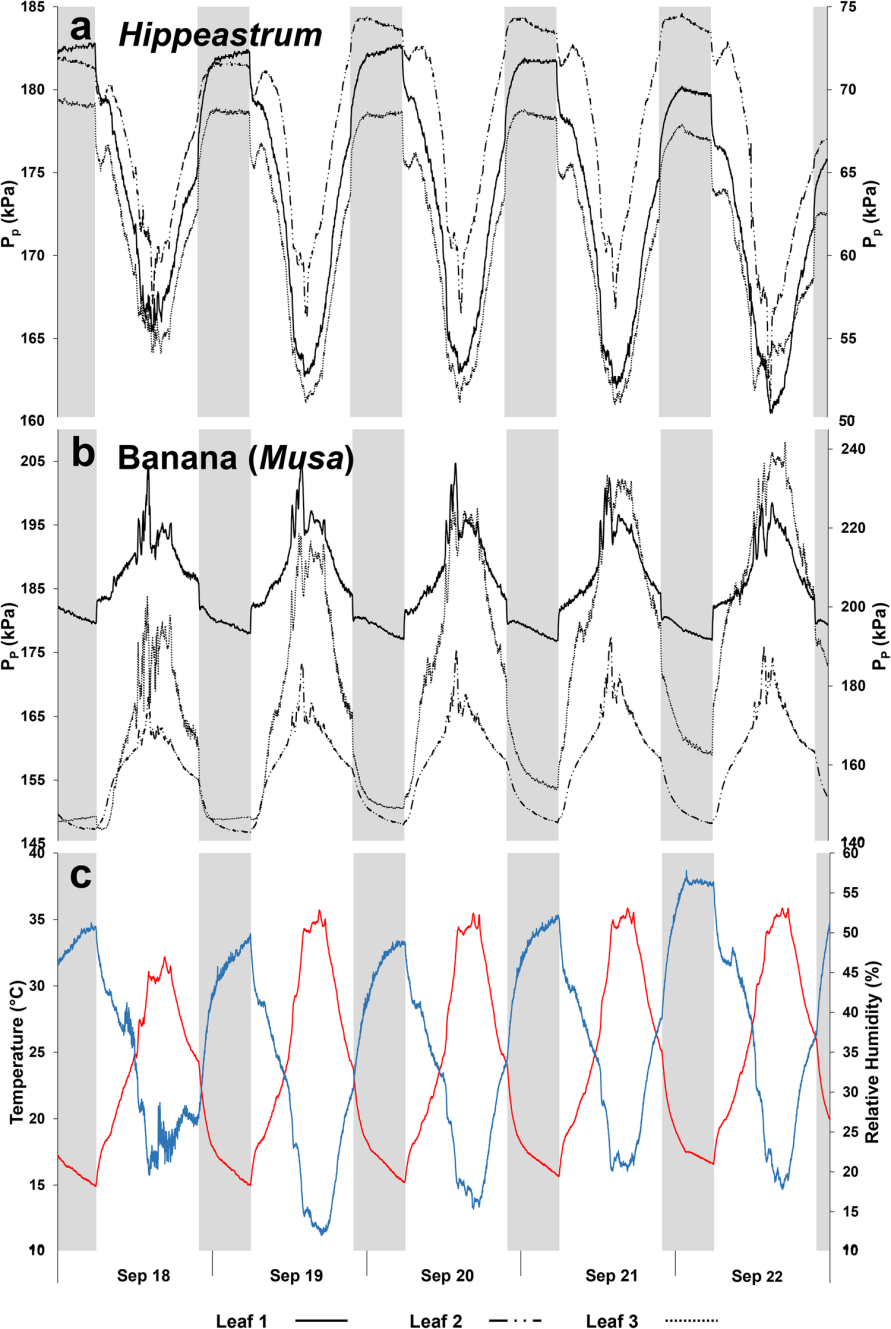
Diel changes of the leaf patch clamp pressure probe (LPCP) output pressure, *P*_p_. **a** Measurements were made on three leaves of a specimen of *Hippeastrum* ‘Red Lion’, of which the *P*_P_ values of Leaf 2 are referenced to right vertical axis. **b** Measurements on three leaves of a small banana plant, with the *P*_P_ values of Leaf 3 referenced to right vertical axis. **c** The corresponding diel changes of temperature (red line) and relative humidity (blue line). Nocturnal phase (8 h) indicated by the gray shaded periods

Similar to its parent species, the *Hippestrum* ‘Red Lion’ cultivar also presents the aerenchyma lacunae interspersing the vascular bundles, running throughout the leaf (Fig. 1). The lacunae occupy 0.63 ± 0.01 (*n* = 18) of the mesophyll thickness. The output pressure, *P*_p_, in *Hippeastrum* varies opposite to that observed on banana, reaching its minimum (Fig. 2a) while in banana it reaches its maximum (Fig. 2b), when the temperature peaks and the relative humidity reaches its lowest (Fig. 2c). Upon turning on the lights, *P*_p_ in *Hippeastrum* decreases sharply in the first 30–40 min and at a slower rate afterwards, reaching its minimum early in the afternoon. After that, *P*_p_ raises slowly until evening time, so that immediately after lights are turned off, it increases extremely fast in next 30–40 min, almost reaching its maximum at a slower rate not so long afterwards. Equally fast changes in *P*_p_ in response to turning on and off the lights but opposite to that observed on *Hippeastrum* (Fig. 2b) are also observed on banana. These results suggest that the changes in *P*_p_ in both species immediately after turning on and off the lights might be related to the opening and closing of stomata triggered by light, and the expected changes in transpiration, as plants react to light and resume photosynthesis. Nevertheless, the diel pattern of *P*_p_ in *Hippeastrum* seems to contradict its relationship with the leaf turgor pressure, *P*_c_, Eq. (1) suggesting that in these plants the two parameters are not related as in banana.

The changes in the output pressure, *P*_p_, in *Hippeastrum* leaves in the first 60 min right after turning on and turning off the lights can be well described by an exponential function so that a relaxation time constant *τ* can be determined:

16 $$\frac{\partial {P}_{\mathrm{p}}}{\partial t}=-\tau {P}_{\mathrm{p}}.$$

**Fig. 3:**
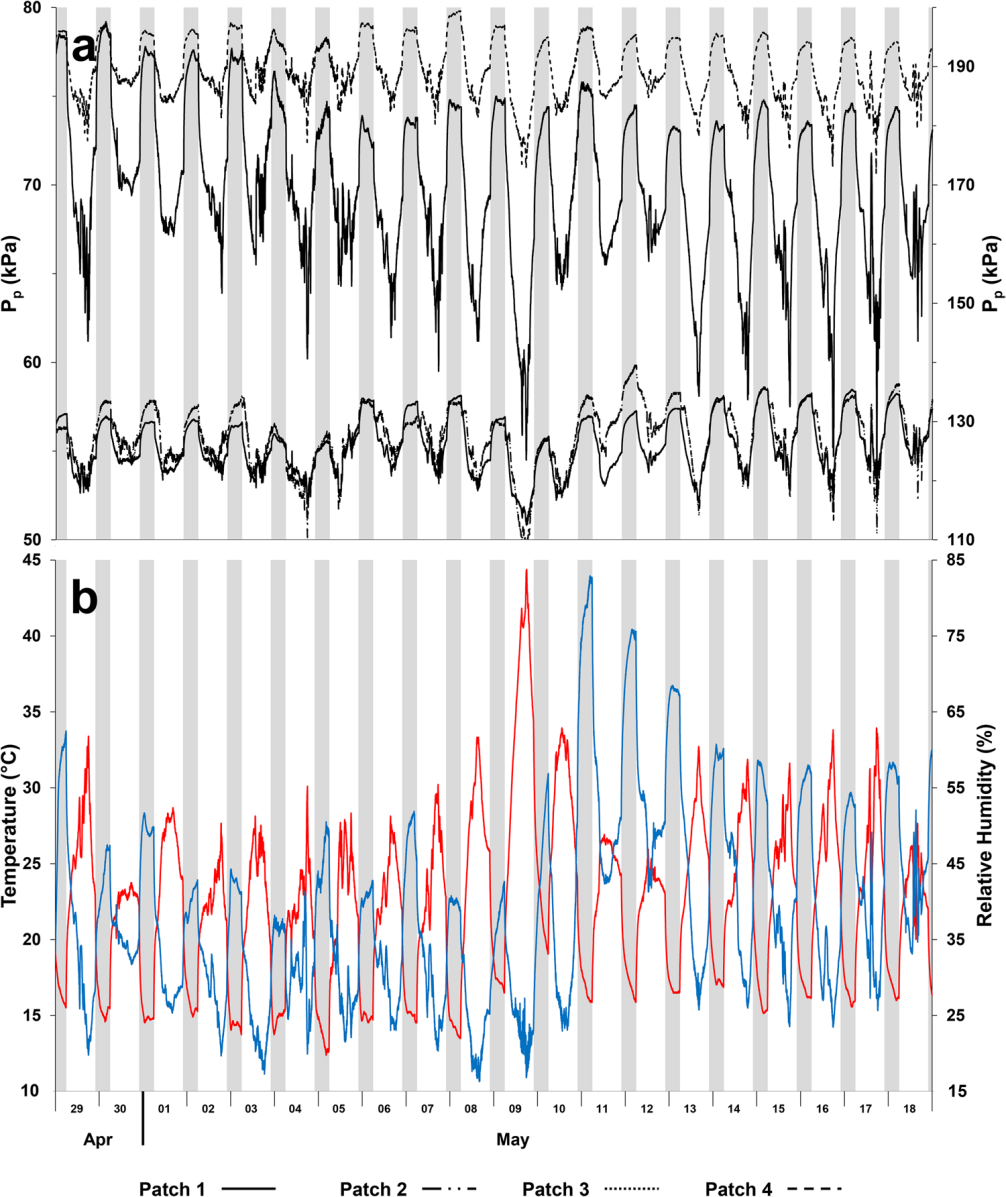
Diel changes of the leaf patch clamp pressure (LPCP) output pressure, *P*_p_, within a mature and fully expanded leaf of a specimen of *Hippeastrum* ‘Red Lion’. **a** *P*_p_ values of four patches applied 10 cm apart and parallel to the midrib along the length of the leaf; values of patches 2 and 3 on the left vertical axis and values of patches 1 and 4 on right vertical axis. **b** The corresponding diel changes of temperature (red line) and relative humidity (blue line). Nocturnal phase (8 h) indicated by the gray shaded periods

One can refer to a relaxation time constant *τ*_on_ after lights are turned on and *P*_p_ decreases, and a relaxation time constant *τ*_off_ after lights are turned off and *P*_p_ increases. Four hours before lights are turned off, the increase in *P*_p_ (Fig. 2a) can be also described by an exponential function with a relaxation time constant *τ*_r_. The differences in *P*_p_ values between leaves of the same plant reflect the contribution of turgor-independent and turgor-dependent factors of different leaf patches in attenuating the applied pressure, *P*_clamp_. This has been observed on both species (Fig. 2). Regardless of the leaf structural symmetry, there is always local differences in the structure (e.g., different ratio of protoplast volume to cell wall, cuticle, and intercellular air spaces) that are easily highlighted when measuring on such a small detection area of 20 mm^2^. The diel pattern and dynamics of *P*_p_ is similar in all leaf patches sensed by different sensors on different leaves of the same plant (Figs. 2a, b). This is also observed between four sensors applied 10 cm apart from each other and parallel to the midrib along the length of the same leaf of a second *Hippeastrum* plant (Fig. 3a). Similar observations were made when applying sensors on the adaxial leaf surface of both *Hippeastrum* plants (results not shown). All the leaf patches responded similarly to changes in the ambient conditions (Fig. 3b). For the period of time covered (Figs. 3a, b), the average relaxation time constants *τ*_on_ = −3.8 ± 0.2 × 10^–4^ min^−1^, *τ*_off_ = 4.3 ± 0.2 × 10^–4^ min^−1^, and *τ*_r_ = 2.0 ± 0.2 × 10^–4^ min^−1^, giving *τ*_r_/*τ*_off_ = 0.47 ± 0.03 (*n* = 80). These results observed on *Hippeastrum* reflect the anatomical symmetry expected for a typical lorate leaf; specifically, the lacunae running the whole leaf parallel to the midrib. The type of cells and their physiology in the different leaf patches must be similar in all of them, so that they all show the same diel pattern of changes of *P*_p_ with time, including their response to the opening and closing of stomata (Fig. 3a). Comparing all the leaves of the same *Hippeastrum* plant for the period shown in Fig. 3a, similar results were obtained, with *τ*_on_ = −4.3 ± 0.3 × 10^–4^ min^−1^, *τ*_off_ = 3.9 ± 0.3 × 10^–4^ min^−1^, and *τ*_r_ = 2.3 ± 0.2 × 10^–4^ min^−1^, giving *τ*_r_/*τ*_off_ = 0.58 ± 0.07 (*n* = 60). The responses of banana upon turning on and off of the lights are faster and in opposite directions than those of *Hippeastrum*. The changes in the banana output pressure, *P*_p_, were best described by Eq. (16) during the first 15 min after lights were turned on and off. Afterwards, *P*_p_ changes slower with time but differently from Eq. (16). Comparing four leaves of the banana plant for the period shown in Fig. 3b, *τ*_on_ = 3.8 ± 0.2 × 10^–3^ min^−1^, *τ*_off_ = −2.6 ± 0.5 × 10^–3^ min^−1^, and *τ*_r_ = -3.4 ± 0.2 × 10^–4^ min^−1^, with *τ*_r_/*τ*_off_ = 0.13 ± 0.01 (*n* = 80). The changes of *P*_p_ in banana are generally faster than in *Hippeastrum*, with *τ*_on_ and *τ*_off_ being 1 order of magnitude higher than that of *Hippeastrum*, making *τ*_r_/*τ*_off_ smaller in banana.

### The dynamics of the output pressure, *P*_p_, of *Hippeastrum* leaves reflects the changes in the leaf aerenchyma pressure, *P*_a_

**Fig. 4:**
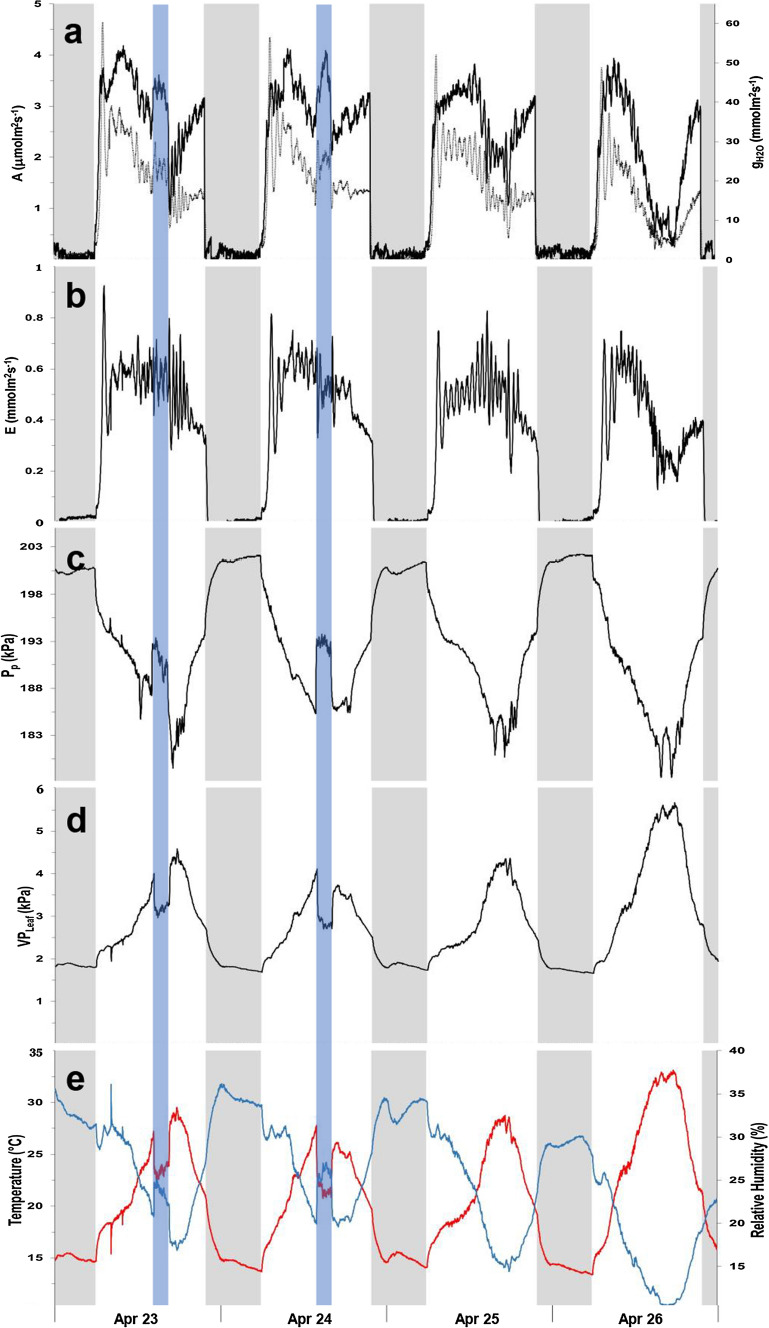
Photosynthetic activity and diel changes of the leaf patch clamp pressure (LPCP) output pressure, *P*_p_, in *Hippeastrum*. **a** Assimilation rate, A (black line), and stomatal water vapor conductance, g_H2O_, (black dotted line). **b** Transpiration rate, E. **c** LPCP output pressure, *P*_p_. **d** Leaf aerenchyma vapor pressure, VP_Leaf_. **e** Temperature (red line) and relative humidity (blue line) measured simultaneously. Ambient temperature was lowered for 2 h as indicated by the periods shaded in blue on April 23 and 24. Nocturnal phase (8 h) indicated by the remaining shaded periods in gray

**Fig. 5:**
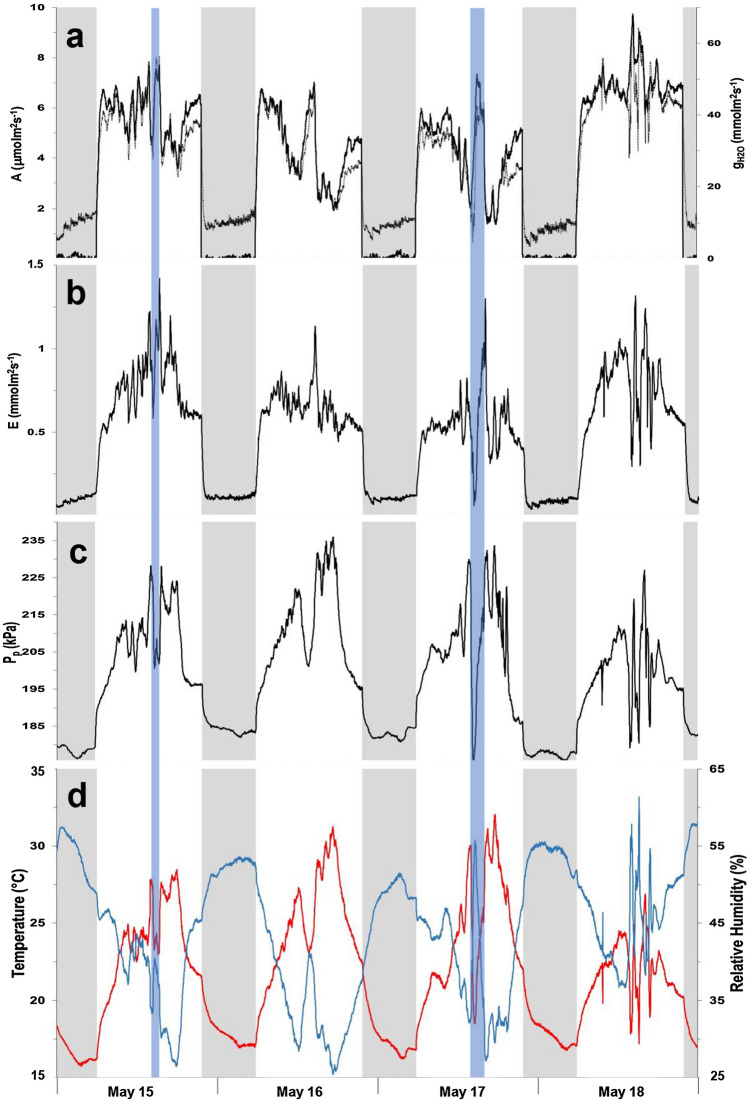
Photosynthetic activity and diel changes of the leaf patch clamp pressure (LPCP) output pressure, *P*_p_, in banana. **a** Assimilation rate, A (black line), and stomatal water vapor conductance, g_H2O_ (black dotted line). **b** Transpiration rate, E. **c** LPCP output pressure, *P*_p_. **d** Temperature (red line) and relative humidity (blue line) measured simultaneously. Ambient temperature was lowered for 1 and 2 h on May 15 and 17 as indicated by the periods shaded in blue. Nocturnal phase (8 h) indicated by the remaining shaded periods in gray

**Fig. 6:**
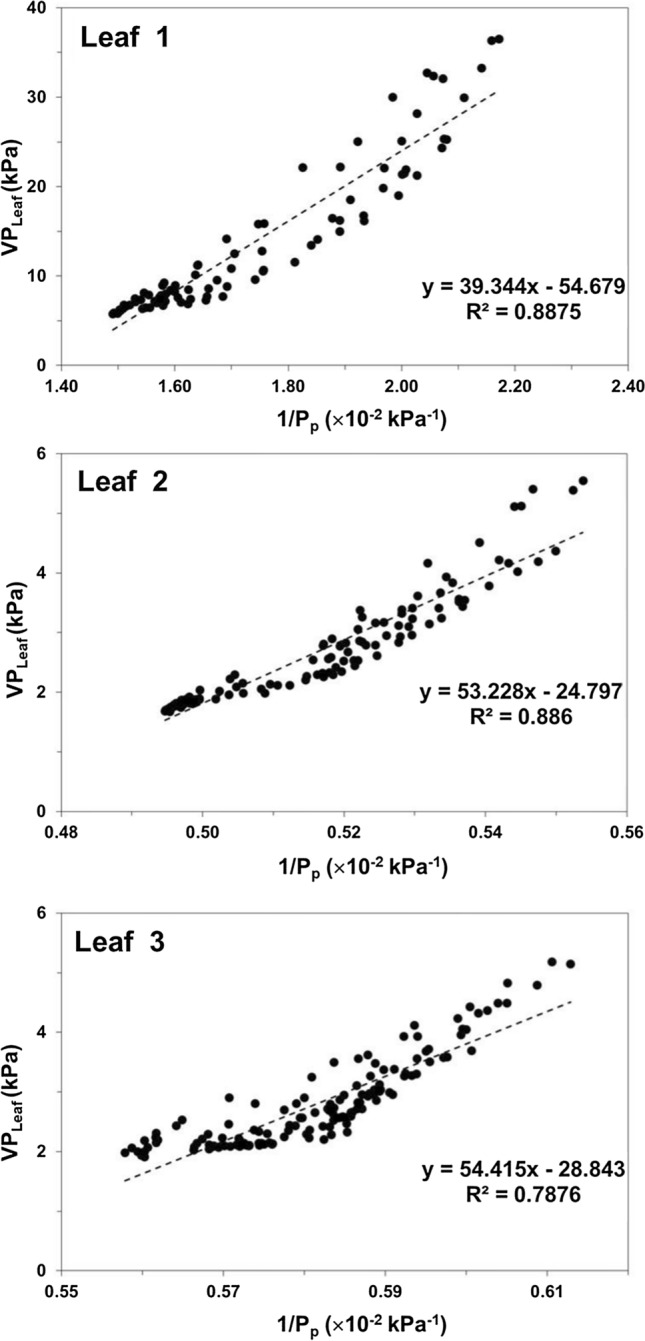
Leaf vapor pressure, VP_Leaf_, vs the leaf patch clam pressure probe (LPCP) output pressure, *P*_p_, in *Hippeastrum*. Leaf vapor pressure measurements were obtained from gas exchange measurements performed simultaneously with LPCP measurements on the same leaf for 64 h on Leaf 1, 113 h on Leaf 2, and 141 h on Leaf 3 of 2 specimens of *Hippeastrum* ‘Red Lion’. Values of the leaf vapor pressure, VP_Leaf_, and the output pressure, *P*_p_, were averaged for 1-h periods

Both species present similar diel patterns of photosynthetic activity, with the photosynthetic parameters showing fluctuations of variable amplitude and frequency (Figs. 4a, b, 5a, b) reflecting the stomatal oscillations, known to occur in plants under unfavorable ambient conditions (Figs. 4e, 5d). In *Hippeastrum*, stomatal oscillations occurred with an average period of 42.2 ± 0.5 min (*n* = 4), while in banana they occurred every 47.2 ± 2.4 min (*n* = 4). Fluctuations in the output pressure, *P*_p_, were also observed on banana, but with more variations in their amplitude (Fig. 5c). These were observed during 10.2 ± 0.5 h (*n* = 4) with a period of 49.2 ± 1.1 min (*n* = 4), when the relative humidity remained below 40% (Fig. 5d). The fluctuations in *P*_p_ in banana occurred with a pattern similar to that of photosynthesis (Fig. 5a, b). Despite the occurrence of occasional fluctuations in P_p_ in *Hippeastrum* (Fig. 4c), their irregular pattern did not resemble that of its photosynthetic activity (Fig. 4a, b). Instead, in *Hippeastrum*, when one compares the pattern of *P*_p_ (Fig. 4c) with that of the leaf aerenchyma water vapor pressure, VP_Leaf_, (Fig. 4d), one finds that the former is a scaled mirror function of the latter. A decrease in *P*_p_ reflects a concomitant increase in VP_Leaf_, as predicted by Eqs. (12) and (15). Plotting the leaf aerenchyma vapor pressure, VP_Leaf_, against the inverse of the observed output pressure, *P*_p_^−1^, of three leaves of the two *Hippeastrum* plants (Fig. 6), both parameters vary linearly as predicted by Eq. (15). As the moisture holding capacity of air increases with temperature, more water evaporates from the thin aqueous film lining the mesophyll cell walls in contact with the intercellular air spaces and the lacunae. Consequently, the leaf aerenchyma water vapor pressure, VP_Leaf_, (Fig. 4d) and the aerenchyma air pressure, *P*_a_, increase Eq. (14). Simultaneously, the output pressure, *P*_p_, will decrease (Fig. 4c), Eqs. (12) and (15), as the leaf aerenchyma air pressure, *P*_a_, and vapor pressure, VP_Leaf_, increase (Fig. 4d). In *Hippeastrum*, *P*_p_ follows the diel changes in *P*_a_ reversely; reaching its minimum when the transpiration rate is at its maximum, and the relative humidity reaches its lowest. Afterwards, *P*_p_ increases back to its maximum range during the dark period, while VP_Leaf_ and *P*_a_ attain their lowest. During the dark period, there is very little change in *P*_p_ in *Hippeastrum* leaves compared to that observed during the light period (Figs. 2a, 4c). With stomata closed (Fig. 4a), the driving force to change *P*_a_ is practically non-existent. Therefore, *P*_p_ will change very little (Figs. 1a, 4c). This stability of the output pressure, *P*_p_, is less observable on banana (Figs. 2b, 5c). Being directly related to the leaf turgor pressure, *P*_c_, Eq. (1), *P*_p_ in banana is, thus, dependent on the redistribution of water between the different regions of the plant body, while recovering the turgor pressure status after a photosynthetic cycle, which depends on the water availability within the tissues. These processes depend on the balance between the amount of water being lost during transpiration and the soil water availability. Therefore, *P*_c_ and, consequently, *P*_p_, in banana change during the dark period between diel photosynthetic cycles (e.g., Leaf 3 of Fig. 2b, Fig. 5c).

Equations (12) and (15) can be tested using the dependence of the leaf gas exchange processes on temperature. By decreasing temperature, the relative humidity of the air will increase (Fig. 4e) due to the decrease of its moisture holding capacity. Consequently, VP_Leaf_ decreases (Fig. 4d), decreasing VPD and creating more favorable and less stressful conditions for plants to photosynthesize. The balance between transpiration demand and water availability in the soil is achieved less costly. This result is shown by the response of the *Hippeastrum* plant on increasing its photosynthesis (Fig. 4a) as temperature decreases (Fig. 4e). Leaf aerenchyma pressure, *P*_a_, will, thus, decrease increasing the output pressure, *P*_p_, in *Hippeastrum* (Fig. 4c), Eq. (12). Increasing the temperature back to its previous levels, all the changes in the different parameters described before will be reversed. Submitting the banana plant to a similar treatment (Fig. 5d), its response in terms of photosynthesis (Fig. 4a) was similar to that observed on *Hippeastrum* (Fig. 4a), but the change observed on *P*_p_ (Fig. 5c) was opposite to that observed on *Hippeastrum* (Fig. 4c), as it decreased with the temperature. This result agrees with the increase in the leaf turgor pressure expected from decreasing VPD by decreasing the temperature and increasing the relative humidity (Fig. 5d). Consequently, as the plant restores and maintains a higher water status, there is a consequent increase in *P*_c_ and *P*_p_ should, thus, decrease Eq. (1) and (Fig. 5c).

## Discussion

### Leaf anatomy determines the origin of the leaf patch clamp output pressure, ***P***_p_

Zimmermann et al. (2010) showed that the diel changes observed on the output pressure, *P*_p_, in banana were reversed to those observed on the leaf turgor pressure, *P*_c_, Eq. (1), as also observed on other species (Zimmermann et al. 2008; Westhoff et al. 2009; Ehrenberger et al. 2012a). Therefore, the output pressure, *P*_p_, in banana leaves raises sharply when lights are turned on (Fig. 2b), triggering stomata to open and the plant to photosynthesize and transpire, causing turgor pressure to decrease. When lights are turned off, the stomata close, ceasing transpiration, and the leaf turgor pressure recovers, causing the output pressure, *P*_p_, to decrease and reach its minimum around dawn the next day, before lights are turned back on again Eq. (1). The fast changes in P_p_ observed on banana, hence in the leaf turgor pressure, which happen during first 15 min immediately after turning on and shutting down the lights reflect the prompt response of stomata in opening and closing, determining transpiration, and thus changing turgor pressure. This result contrasts with observations of the output pressure, *P*_p_, on plants growing on the field where the gradual opening and closing of stomata, following sunlight, and the subsequent photosynthetic rate and changes in turgor pressure cause smoother diel patterns of the output pressure *P*_p_ (Westhoff et al. 2009; Rüger et al. 2010; Fernández et al. 2011; Ehrenberger et al. 2012a, b). Both these situations illustrate the usefulness of using the LPCP to study other physiological processes occurring in leaves indirectly, while looking at the changes in turgor pressure and plant water status non-invasively and continuously.

The species studied in this work present similar diel patterns of photosynthetic activity (Figs. 4a, 5a), suggesting that changes in the leaf turgor pressure due to transpiration should occur in the same direction in both. That is, the leaf turgor pressure decreases during the day and recovers back to its previous level during the night. However, the output pressure, *P*_p_, diel pattern differs remarkably between both species (Figs. 2a, b). These results illustrate quite well the importance of knowing leaf anatomy upon interpreting LPCP results. The different composition and proportion of the leaf components is the main reason behind the differences observed between both species. The presence of big aerenchyma lacunae filled with saturated air composing most of the mesophyll in *Hippeastrum* leaves (Fig. 1) makes the LPCP sensor to detect changes in the aerenchyma air pressure, *P*_a_, Eq. (12) (Figs. 2a, 3a, 4b) instead of the mesophyll turgor pressure, *P*_c_, Eq. (1) as observed on banana (Figs. 2b, 5b) and other species (e.g., Zimmerman et al. 2008, 2013; Rüger et al. 2010; Fernández et al. 2011; Ehrenberger et al. 2012a; Bramley et al. 2013). In *Hippeastrum* leaves the much greater proportion of aerenchyma compared to chlorenchyma makes changes in leaf patch chlorenchyma turgor pressure undetectable by the LPC sensor, according to the model Eqs. (12) and (15) and confirmed experimentally (Fig. 6). The ratio of chlorenchyma to leaf intercellular air spaces in banana is much greater than that observed on *Hippeastrum* plants, making the changes in the leaf intercellular air spaces pressure negligible in contributing to LPCP measurements under normal physiological conditions (Zimmermann et al. 2008, 2010; Ehrenberger et al. 2012b).

Despite the different parameters, relating to different physiological processes, sensed by the LPCP probe, the output pressure, *P*_p_, can be, nevertheless, similar in both species (Figs. 2a, b, 4c, 5c). However, their dynamics and, specifically, those changes triggered by the plants responses to light are quite different. The relaxation time constants *τ*_on_ and *τ*_off_ of banana are 10 times bigger than those of *Hippeastrum*, while the output pressure, *P*_p_, changes slightly faster in banana in the evening, as indicated by its relaxation time constant *τ*_r_. These differences in how the output pressure, *P*_p_, changes with time are explained by the physiological nature of the parameters being sensed on both species. The leaf aerenchyma air pressure, *P*_a_, in *Hippeastrum* is highly dependent on the temperature that affects the leaf-to-air vapor pressure deficit, VPD, which drives transpiration. Once transpiration stops by the closing of stomata in *Hippeastrum*, the decrease of the leaf aerenchyma pressure, *P*_a_, reflected in the recovery of the output pressure, *P*_p_, depends on the effect of the decrease in temperature on decreasing the moisture holding capacity of air, i.e., aerenchyma vapor pressure, VP_Leaf_, inside leaves. After lights are turned off, less water will evaporate from the thin aqueous film lining the mesophyll cell walls facing the leaf intercellular air spaces and lacunae in *Hippeastrum*, as the moisture holding capacity of air decreases with temperature. Under slower decreasing temperature after lights are turned off, so will the decrease the leaf aerenchyma pressure, *P*_a_, and the consequent increase the output pressure, *P*_p_, also be slower.

After lights are turned off, the decrease in the output pressure, *P*_p_, in banana leaves, reflects the recovery in the leaf turgor pressor pressure, *P*_c_, once transpiration stops. The simultaneous recovery of turgor pressure in the leaf patch mesophyll cells of banana is much faster, sensed by output pressure, *P*_p_, Eq. (1), comparing with the decrease in the leaf aerenchyma pressure, *P*_a_, in *Hippeastrum* that depends on the temperature; thus, not controlled by the plant. At night, all the leaf patch mesophyll cells in banana will recover its turgor pressure simultaneously to a water potential level that depends on the overall water balance in the plant body after each photosynthetic cycle. In *Hippeastrum* plants under the same conditions, the decrease in the leaf patch aerenchyma pressure, *P*_a_, and the concomitant recovery of the output pressure, *P*_p_, do not depend directly on the redistribution of water in the plant body, rather on how fast the temperature changes. This could also explain the higher value of the relaxation time constant *τ*_r_ of banana during late evening, while the changes in the ambient parameters create more favorable conditions. The difference in the origin of the output pressure, *P*_p_, between both species also explains the less variability of *P*_p_ in *Hippeastrum* plants during the night, when the temperature changes are much smaller than during the light period. In banana, changes in the leaf patch turgor pressure are then expected during the night (Figs. 1, 2) due to their dependence on the redistribution of water in plant body after each photosynthetic cycle, which depends on the overall plant–soil–atmosphere water balance.

### The physiological role of leaf aerenchyma in *Hippeastrum* plants and its advantages in unfavorable environments

*Hippeastrum* plants are found mainly in tropical and subtropical habitats with two dominant seasons, wet and dry, throughout the year, with many species living in underbrush or exposed to full sun, including transient flood areas and dry habitats (Alves-Araújo et al. 2005; Alves-Araújo 2007). They must cope with transient extreme drought or anoxic conditions that can last for long periods. Therefore, the presence of leaf aerenchyma and a bulbous form allowing water and nutrient storage seem advantageous. Intercellular air spaces within plants have many functions (Raven 1996), but in vascular land plants, they are mostly used for gas distribution (CO_2_ and O_2_) within the plant body (Armstrong 1980; Armstrong et al. 1994; Parkhurst 1994; Raven 1996). The presence of comparatively large areas of intercellular air spaces within leaves also increases the light path through them, increasing the likelihood of photons encountering pigments and promoting photosynthesis. This is achieved by providing a relatively big internal boundary between two phases with large differences in the refractive index (Vogelmann 1993; Raven 1996). Light scattering is an inevitable consequence of leaf intercellular air spaces. An almost twofold increase in the photosynthetic rate was observed in plants with a relatively large leaf intercellular air space-cell wall boundary that promoted light scattering (De Lucia et al. 1996). Having a large fleshy tunicate bulb and big specialized leaf aerenchyma seems to provide *Hippeastrum* plants advantages while living under such conditions. On one hand, a reserve bulbous organ for storage of nutrients and water allows them to survive prolonged drought, even more efficiently when these plants are hysteranthous (Arroyo and Cutler 1984; Meerow 2004, 2009). On the other hand, the big symmetrically distributed leaf aerenchyma lacunae allow access of air, particularly oxygen, to the bulb and roots, especially when plants are forced to live in areas that are regularly flooded for long periods, facing anoxia eventually. Additionally, the leaf aerenchyma lacunae seem particularly advantageous by promoting light scattering and increasing its reach within leaves, thus promoting photosynthesis. Considering the stomata distribution in *Hippeastrum* leaves, although amphystomatous, with higher density of sunken stomata on the abaxial surface (Zhou et al. 2012; Marques 2015), all these adaptions seem to make *Hippeastrum* plants specifically adapted to live under extreme environmental opposites. Looking at the diel changes in the aerenchyma air pressure, *P*_a_, sensed by the output pressure, *P*_p_, in *Hippeastrum* plants (Fig. 4c), the stomatal oscillations observed (Fig. 4a, b) seemed particularly useful in keeping a low transpiration rate (Fig. 4b) under such stressful environmental conditions, namely, a very low relative humidity (Fig. 4e). In comparison, the banana plant showed stomatal oscillations and similar transpiration rate (Fig. 5a) under higher relative humidity (Fig. 5d), i.e., under lower leaf-to-air vapor pressure deficit, VPD, conditions.

## Conclusion

The leaf patch clamp pressure probe is a non-invasive plant-based system that measures the output pressure, *P*_p_, in response to the pressure applied by two magnets clamped to a leaf. In many plant species, the diel pattern of the output pressure, *P*_p_, follows the changes in the leaf turgor pressure, *P*_c_, reversely. However, when applied on species in which the aerenchyma constitutes most of the mesophyll volume, e.g., *Hippeastrum*, the changes in the output pressure, *P*_p_, reflect the changes in the leaf aerenchyma air pressure, *P*_a_. Therefore, the dependence of the leaf aerenchyma air pressure, *P*_a_, on the gas exchange process that occur during photosynthesis allows an indirect assessment of turgor-dependent processes as well as factors affecting turgor regulation and plant–water relations through non-invasive measurements by the LPCP method.

### *Author contribution statement*

The author confirms sole responsibility for the following: study conception and design, data collection, analysis and interpretation of results, and manuscript preparation.

## Data Availability

The datasets generated during and/or analyzed during the current study are available from the corresponding author on reasonable request.

## Abbreviations

*ε*: Volumetric elastic modulus
F_a_: Attenuation factor
LPCP: Leaf patch clamp pressure probe
*P*: Pressure
*P*_a_: Aerenchyma pressure
*P*_c_: Turgor pressure
*P*_clamp_: Pressure applied by magnets
*P*_in_: Input pressure
*P*_p_: Output pressure
P_sat_: Saturated air pressure
*ξ*: Sum of the all air components partial pressures except water
*T*: Temperature
*T*_f_: Transfer function
*τ*_on_: Relaxation time constant after lights turned on
*τ*_off_: Relaxation time constant after lights turned off
*τ*_r_: Relaxation time constant during evening before lights are turned off
*V*: Volume
*V*_a_: Leaf patch aerenchyma volume
VP_Leaf_: Aerenchyma vapor pressure
VPD: Leaf-to-air vapor pressure deficit

## Footnotes

1 In fact, as the air within the leaf intercellular air spaces is in water potential equilibrium (-3.0 to -0.05 MPa) with the cell wall surfaces of the cells lining them, the relative humidity of the leaf internal atmosphere varies between 98 and 100% (Salisbury and Ross 1991).
